# Circulatory Exosomes from COVID-19 Patients Trigger NLRP3 Inflammasome in Endothelial Cells

**DOI:** 10.1128/mbio.00951-22

**Published:** 2022-05-19

**Authors:** Subhayan Sur, Robert Steele, T. Scott Isbell, Ranjit Ray, Ratna B. Ray

**Affiliations:** a Departments of Pathology, Saint Louis University, St. Louis, Missouri, USA; b Internal Medicine, Saint Louis University, St. Louis, Missouri, USA; Johns Hopkins University School of Medicine; Johns Hopkins Bloomberg School of Public Health

**Keywords:** SARS-CoV-2, exosomes, NLRP3, inflammasome, endothelial cells, COVID-19

## Abstract

Severe acute respiratory syndrome coronavirus 2 (SARS-CoV-2) infection induces inflammatory response, cytokine storm, venous thromboembolism, coagulopathy, and multiple organ damage. Resting endothelial cells prevent coagulation, control blood flow, and inhibit inflammation. However, it remains unknown how SARS-CoV-2 induces strong molecular signals in distant cells for immunopathogenesis. In this study, we examined the consequence of human endothelial cells, microvascular endothelial cells (HMEC-1), and liver endothelial cells (TMNK-1) to exosomes isolated from plasma of mild or severe COVID-19 patients. We observed a significant induction of NLRP3, caspase-1, and interleukin-1β (IL-1β) mRNA expression in endothelial cells following exposure to exosomes from severe COVID-19 patients compared with that from patients with mild disease or healthy donors. Activation of caspase-1 was noted in the endothelial cell culture medium following exposure to the COVID-19 exosomes. Furthermore, COVID-19 exosomes significantly induced mature IL-1β secretion in both HMEC-1 and TMNK-1 endothelial cell culture medium. Thus, our results demonstrated for the first time that exosomes from COVID-19 plasma trigger NLRP3 inflammasome in endothelial cells of distant organs resulting in IL-1β secretion and inflammatory response.

## OBSERVATION

SARS-CoV-2 infection, viral pathogenesis, and resulting fatal multiorgan damage are global health concerns. The SARS-CoV-2 envelop spike protein interacts with angiotensin-converting enzyme 2, which is present on many cell surfaces as a receptor, but lung epithelial cells are probably the most susceptible cells for virus entry and replication causing human disease. Clinical observations indicate that severely ill COVID-19 patients experience chronic inflammation, cytokine storm, venous thromboembolic, coagulopathy, and the development of extrapulmonary tissue/organ dysfunctions. The pathophysiology of these diverse manifestations is not clear but is thought to occur in part to a dysregulated inflammatory response of immune and endothelial cells.

The innate immune system is the first line of defense by which the human body recognizes and eliminates foreign pathogenic infection through the involvement of highly conserved sensors, called pattern recognition receptors (PRRs). Inflammasomes, a high-molecular weight cytoplasmic multiprotein complex of sensor protein and inflammatory caspase, play a crucial role in sensing the external stimuli as well as in inducing a cellular response. The NLR family pyrin domain-containing 3 (NLRP3) is a type of PRR and is the most extensively studied inflammasome responsible for inflammation and antiviral responses (1). The NLRP3 inflammasome recruits pro-caspase-1 and activates caspase-1 by proteolytic cleavage resulting in caspase-1-dependent proteolytic maturation and secretion of interleukin 1β (IL-1β) (2). A range of stimuli during pathogenic infections, tissue damage, or metabolic imbalances activate NF-κB which transactivates various effector genes, including NLRP3, and pro-IL-1β (1).

Aberrant activation of the NLRP3 inflammasome or chronic inflammation triggers cellular damage resulting in severe pathological injury (1). Viral infection causes chronic inflammation and triggers NLRP3 inflammasome activation in immune cells. The hepatitis C virus (HCV), HCV core protein, SARS-CoV viroporin, influenza virus M2, or encephalomyocarditis virus viroporin 2B induces NLRP3 inflammasome activation (3–5). The non-immune cells, such as epithelial cells, endothelial cells, and fibroblasts, also contribute to innate immunity (6). Hyperinflammation, venous thromboembolic, dysregulated blood clotting, and multiple organ damage in severely ill COVID-19 patients are suggested cause a to dysfunction of endothelial cells (7), and a careful investigation is needed for understanding the molecular mechanism of actions.

Exosomes are extracellular vesicles (30 to 150 nm) and are formed by the interior budding of endosomal membranes to form large multivesicular bodies. Exosomes play an important role in cellular communication and disease pathogenesis. Exosomes are also involved in viral spread, immune regulation, and antiviral response during infection (8, 9). We have reported previously that exosomes released from HCV-infected hepatocytes enhance fibrogenic markers in hepatic stellate cells (10). SARS-CoV-2 is not known to cause viremia, and clinical data indicate that virus-infected individuals show other organ abnormalities during infection. The functional role of exosomes from SARS-CoV-2-infected cells in distant organs for pathogenic consequences remains unknown. An active NLRP3 inflammasome in peripheral blood mononuclear cells (PBMCs) and tissues of postmortem patients upon autopsy from COVID-19 patients was also reported (11), although a supporting mechanism for the observation was not well defined. SARS-CoV-2 directly or indirectly triggers inflammasomes, leading to the secretion of pleiotropic IL-1 family cytokines (IL-1β and IL-18) (12), although the molecular mechanisms for COVID-19 disease pathogenesis remain poorly understood.

We recently found that tenascin-C and fibrinogen-β are highly abundant in exosomes from COVID-19 patient plasma samples (13). Subsequently, we showed that exosomes from COVID-19 patients trigger inflammatory signals to hepatocytes by inducing NF-κB through tenascin-C and fibrinogen-β. Thus, we hypothesized that exosomes from COVID-19 patients may influence endothelial cell dysfunction and inflammatory response at distant organs. In this study, we found that exosomes from COVID-19 patients stimulate NLRP3 inflammasome formation and IL-1β production in endothelial cells and may promote a systemic inflammatory response.

### Exosomes from COVID-19 patients trigger inflammasome genes in endothelial cells.

**FIG 1:**
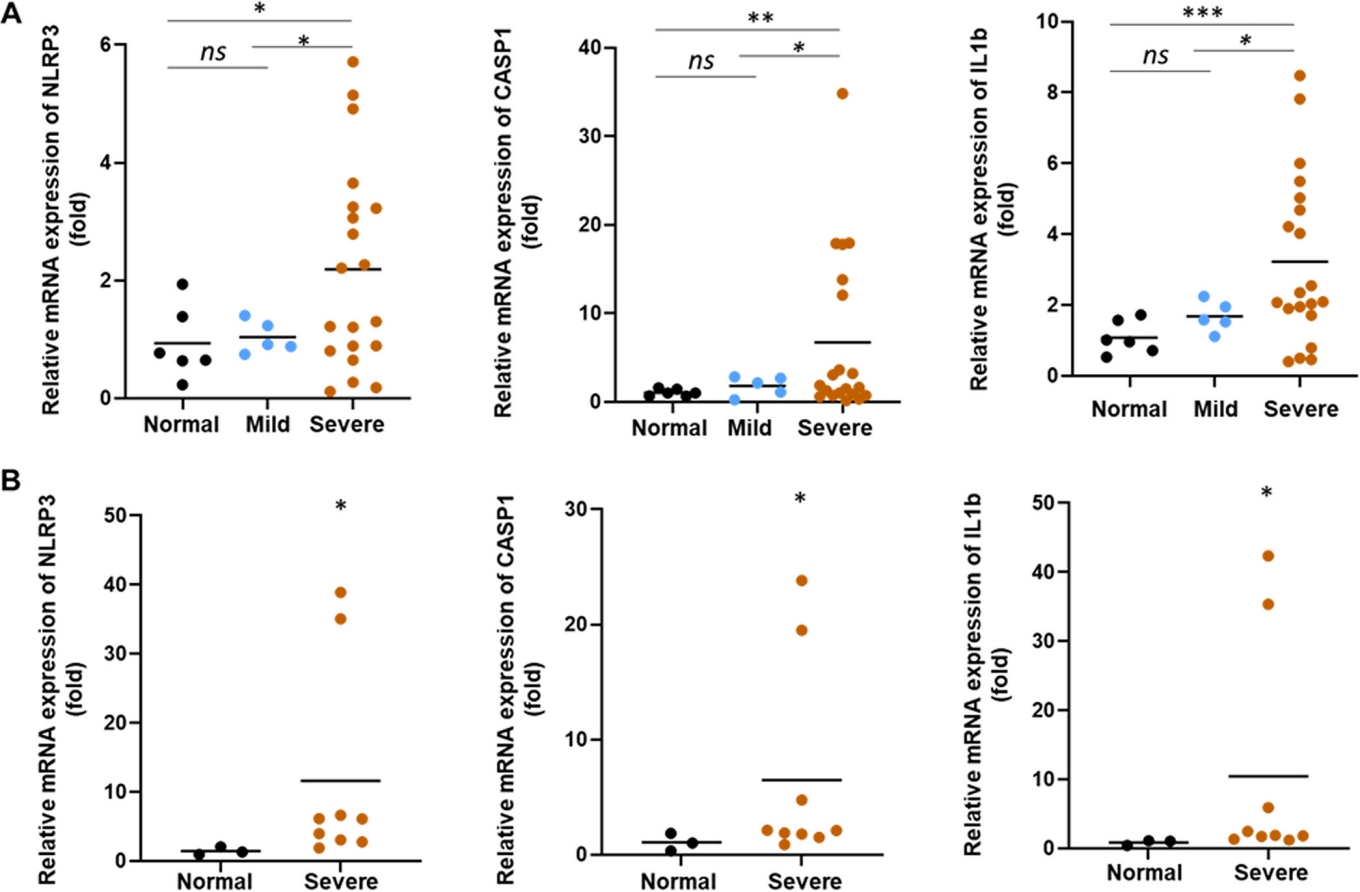
Exosomes from COVID-19 patients induce inflammasome genes. HMEC-1 (A) and TMNK-1 (B) cells were exposed to exosomes isolated from healthy subjects (normal) and patients with mild COVID-19 symptoms or severe COVID-19 patients for 48 h. Total RNA was isolated, and the relative mRNA expression of NLRP3, caspase-1 (CASP1), and 1L-1β was measured by reverse transcription-quantitative PCR (qRT-PCR). 18S rRNA was used as an internal control. The results are presented as dot plots. The line indicates the mean value per group. Fold regulation is expressed as the 2^(−ΔΔ^*^CT^*^)^ method. (ns, not significant; *, *P* < 0.05; **, *P* < 0.01; ***, *P* < 0.001).

COVID-19 patients were admitted to the intensive care unit (ICU) in our academic medical center, and samples were collected on the day of their arrival. The exosomes from the plasma of healthy subjects (*n* = 6) and mild COVID-19 (*n* = 5) and severe COVID-19 patients (*n* = 20) were isolated. The exosomes were characterized by the expression of CD63 and TSG101 by Western blot analysis and further confirmed by transmission electron microscopy as described previously (13). To know the effect of COVID-19 exosomes on inflammasome formation in human endothelial cells, HMEC-1 (human foreskin origin) or TMNK-1 (liver origin) cells were treated with normal or COVID-19 exosomes. We observed a significant increase in mRNA expression of NLRP3, pro-caspase-1 (CASP1), and pro-IL-1β genes in HMEC-1 cells exposed to exosomes from the plasma of severe COVID-19 patients compared with that from healthy subjects (Fig. 1A). Similar observations were noted with TMNK-1 cells following COVID-19 exosome exposure (Fig. 1B). The results indicate that COVID-19 exosomes transcriptionally induce NLRP3 inflammasome components in endothelial cells of two different anatomical sources. We similarly incubated THP-1 cells (a human cell line derived from an acute monocytic leukemia patient, with or without phorbol myristate acetate (PMA) treatment) with exosomes from plasma of COVID-19 patients or those of healthy subjects, and a detectable change of NLRP3 inflammasome signaling change was not observed (see Fig. S1 in the supplemental material). Interestingly, we observed exosomes from mild patients, when treated with endothelial cells, did not induce inflammasomes. Clinical data of the liver enzyme level of COVID-19 patients (Table 1) did not correlate with inflammasome induction in either endothelial cell line.

**TABLE 1:** Liver enzyme levels

Sample name by disease severity	Levels (U/liter) of:	
	Alanine transaminase	Aspartate transferase (U/liter)
Severe symptoms		
1	252	193
2	14	13
3	83	82
4	ND^*a*^	ND
5	17	19
6	15	37
7	59	27
8	62	48
9	20	21
10	19	28
11	33	28
12	21	44
13	47	64
14	40	99
15	8	15
16	12	31
17	19	47
18	63	56
19	26	25
20	84	108
Mild symptoms		
M1	35	46
M2	17	19
M3	17	34
M4	16	25
M5	6	15

a ND, not done.

10.1128/mbio.00951-22.1 FIG S1 Exosomes from COVID-19 patients did not induce IL-1β mRNA expression in THP1 cells. Cells were exposed to exosomes isolated from healthy subjects (*n* = 6) (referred to as normal) or severe COVID-19 (*n* = 12) patients for 48 h. Total RNA was isolated, and relative mRNA expression of IL-1β was measured by qRT-PCR. 18S rRNA was used as an internal control. Download FIG S1, TIF file, 0.1 MB. Copyright © 2022 Sur et al. 2022 Sur et al. https://creativecommons.org/licenses/by/4.0/ This content is distributed under the terms of the Creative Commons Attribution 4.0 International license.

### Exosomes from COVID-19 patients activate caspase-1 in endothelial cells.

**FIG 2:**
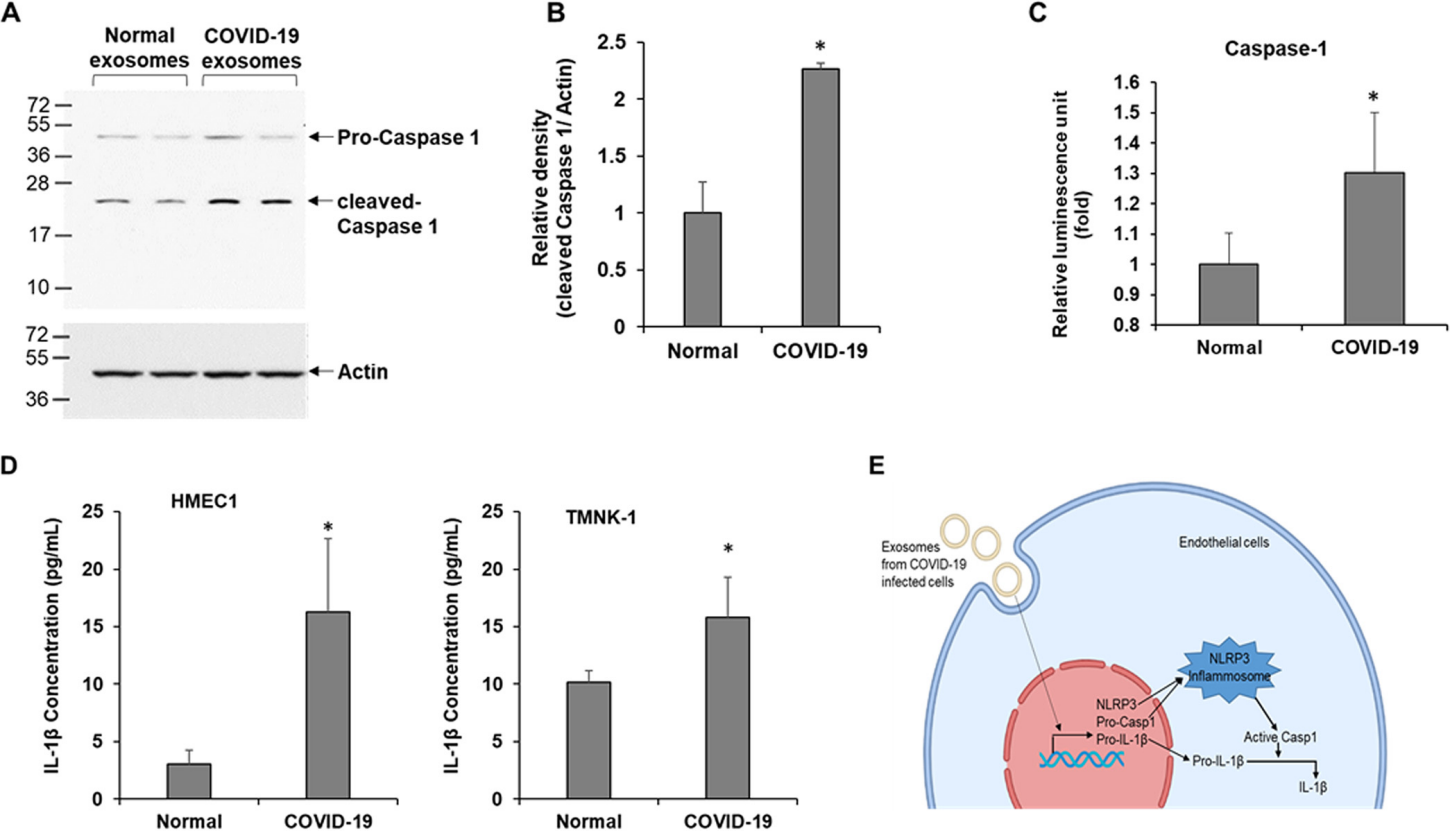
Exosomes isolated from COVID-19 patients activate caspase-1 and induce 1L-1β secretion. (A) HMEC-1 cells were exposed to exosomes from normal and COVID-19 patients for 48 h, and cell lysates were subjected to Western blot analysis for caspase-1 using a specific antibody. The membrane was reprobed for actin as an internal control. (B) The quantitative presentation of band intensities using Image J software is shown on the right. (C) Caspase-1 activity was measured in exosomes exposed to HMEC-1 culture medium using the Caspase-Glo 1 inflammasome assay reagent. Luminescence was read after 3 h of incubation with the reagent, and results are presented as relative luminescence unit. (D) HMEC-1 or TMNK-1 cells were exposed to exosomes from normal and COVID-19 patients for 48 h, and IL-1β from culture medium was assayed using the ELISA Max deluxe set human IL-1β kit. Relative absorbance was measured at 450 nm. The concentration of IL-1β in the medium was calculated from a standard curve. The small bar indicates standard error (*, *P* < 0.05). (E) The schematic presentation shows exosomes secreted from SARS-CoV-2-infected cells that trigger NLRP3, pro-caspase-1 (Casp1), and pro-IL-1β transcription resulting in the activation of Casp1 followed by IL-1β via the NLRP3 inflammasome in endothelial cells.

The NLRP3 inflammasome is formed by oligomerization of NLRP3 proteins in the cytoplasm (4). A functionally active inflammasome recruits and activates pro-caspase-1 by proteolytic cleavage. We examined caspase-1 expression in endothelial cells following exposure to COVID-19 exosomes. A Western blot analysis revealed a significant increase of cleaved caspase-1 in severe COVID-19 exosome-exposed HMEC-1 cells as that compared with exosomes from normal healthy subjects (Fig. 2A and B). Caspase-1 is relatively stable and is released from inflammasome -activated cells (14). We measured active caspase-1 in the culture medium of exosome-exposed endothelial cells and a significantly high caspase-1 activity was detected (Fig. 2C).

### Exosomes from COVID-19 patients induce maturation and secretion of IL-1β in endothelial cells.

Active caspase-1 cleaves pro-IL-1β into its mature and functionally active IL-1 β cytokine. We examined the mature IL-1β level in the culture medium by enzyme-linked immunosorbent assay (ELISA). Exposure of COVID-19 exosomes significantly increased the IL-1β level in the culture medium of HMEC-1 compared with the exosomes from healthy donors treated HMEC1 culture medium (Fig. 2D). A similarly increased IL-1β production was seen in the culture medium of TMNK-1 cells when exposed to COVID-19 exosomes (Fig. 2D). Thus, COVID-19 exosomes appeared to trigger IL-1β production through activation of the NLRP3 inflammasome in both of the endothelial cells (Fig. 2D). We did not find any significant cell death following COVID-19 exosome treatment and did not observe a strong indication of gasdermin D cleavage in exosome exposure from severe COVID-19 patients (see Fig. S2 in the supplemental material).

10.1128/mbio.00951-22.2 FIG S2 Exosomes from COVID-19 patients did not induce gasdermin D cleavage. HMEC-1 cells were exposed to exosomes from normal and COVID-19 patients for 48 h, and cell lysates were subjected to Western blot analysis for gasdermin D using a specific antibody (Sigma; G7422). The membrane was reprobed for actin as an internal control. Download FIG S2, TIF file, 0.2 MB. Copyright © 2022 Sur et al. 2022 Sur et al. https://creativecommons.org/licenses/by/4.0/ This content is distributed under the terms of the Creative Commons Attribution 4.0 International license.

We investigated the effect of exosomes isolated from COVID-19 patients on endothelial cells of two distinct organs. Our results revealed that exosomes from severe COVID-19 patients trigger (i) NLRP3, caspase-1, and IL-1β transcription; (ii) NLRP3 inflammasome activation; and (iii) maturation and secretion of IL-1β from endothelial cells. To our knowledge, this is the first demonstration of a mechanism for the regulation of endothelial cell function (inflammation) by exosomes during severe COVID-19 disease.

Endothelial cells provide a selectively permeable barrier to blood and regulate inflammation, platelet aggregation, thrombosis, and vascular smooth muscle proliferation (15). Resting endothelial cells prevent coagulation, control blood flow and passage of proteins from blood into tissues, and inhibit inflammation. Endothelial cell dysfunction resulting in multiorgan failure is common feature of many viral infections, including influenza-A H1N1, SARS-CoV, Middle East respiratory syndrome (MERS)-CoV, and dengue virus (16, 17). Dengue virus NS1, NS2A, and NS2B proteins also induce the NLRP3 inflammasome, and IL-1β release in endothelial cells results in endothelial cell inflammation and dysfunction (17). Association of COVID-19 disease severity with pulmonary endothelial cell dysfunction with impaired microcirculatory function, including venous thromboembolic disease and multiple organ involvement, are reported (7). The deregulated host inflammatory response and cytokine storm are probably the drivers of COVID-19 severity (18). An elevated level of IL-1β and an association with viral load and severity are also reported from COVID-19 patient blood (18, 19). However, the effect of endothelial cell dysfunction in SARS-CoV-2 infection is not clearly known. A recent study demonstrated that the SARS-CoV-2 N protein activates the NLRP3 inflammasome and induces IL-1β and IL-6 production in monocytes (20). However, we did not detect the SARS-CoV-2 N gene in COVID-19 patient exosomes. In a proteomic analysis by mass spectrometry, we did not detect NLRP3, caspase-1, IL-1β, or NF-κB proteins in the COVID-19 patient exosomes (13). Although the mechanism of NLRP3 inflammasome activation remains unknown in endothelial cells, studies indicated that NF-κB, which is activated upon a range of stimuli during viral infection, transcribes effector genes NLRP3 and pro-IL-1β (1). We showed previously that tenascin-C and fibrinogen-β are highly abundant in exosomes from COVID-19 patients, which activate NF-κB in hepatocytes (13), and may play a role in this process. We also observed that the SARS-CoV-2 spike protein, especially the S1 region, is present in the exosomes from patient plasma. Although the unmodified viral spike protein was used widely as a vaccine, diverse functions of this protein were reported (21, 22). We therefore expressed the SARS-CoV-2 spike protein in different cell lines and isolated exosomes from culture media to incubate with endothelial or THP1 cells. Interestingly, we did not observe inflammasome induction in these cells following exposure to the spike exosomes.

The mechanism of deregulation in multiple organs, including, but not limited to, cardiac, neurologic, hemostatic, kidney, and liver during SARS-CoV-2 infection, is not clear. Exosomes play an important role in cell-to-cell communication and viral pathogenesis. Many RNA viruses utilize the exosomal communication for viral pathogenesis (10, 23). For example, exosomes from hepatitis C virus-infected hepatocytes carry materials for the activation of hepatic stellate cells and induce fibrosis (10). Exosome-mediated regulation of endothelial cells was reported in pregnant women (24). Exosomes from activated monocytes activate human brain microvascular endothelial cells to stimulate cytokines, namely, IL-1β and IL-6, through the induction of NF-κB (25). Thus, exosomes may serve as an important mediator for endothelial cell dysfunction and inflammation in various organs during SARS-CoV-2 pathogenesis. In summary, our results suggested that COVID-19 plasma exosome exposure induces the NLRP3 inflammasome in endothelial cells of distant organs which may be one of the mechanisms of endothelial cell dysfunction and inflammation during severe COVID-19 disease.

The Materials and Methods are provided in Text S1 in the supplemental material.

10.1128/mbio.00951-22.3 TEXT S1 Exosomes from COVID-19 patients did not induce IL-1b mRNA expression in THP1 cells Text S1, PDF file, 0.1 MB. Copyright © 2022 Sur et al. 2022 Sur et al. https://creativecommons.org/licenses/by/4.0/ This content is distributed under the terms of the Creative Commons Attribution 4.0 International license.
