# Supplementary figures and images for: Circulatory Exosomes from COVID-19 Patients Trigger NLRP3 Inflammasome in Endothelial Cells
Source: mBio. 2022 May 19;13(3):e00951-22. doi: 10.1128/mbio.00951-22 (PMC9239151; doi:10.1128/mbio.00951-22)

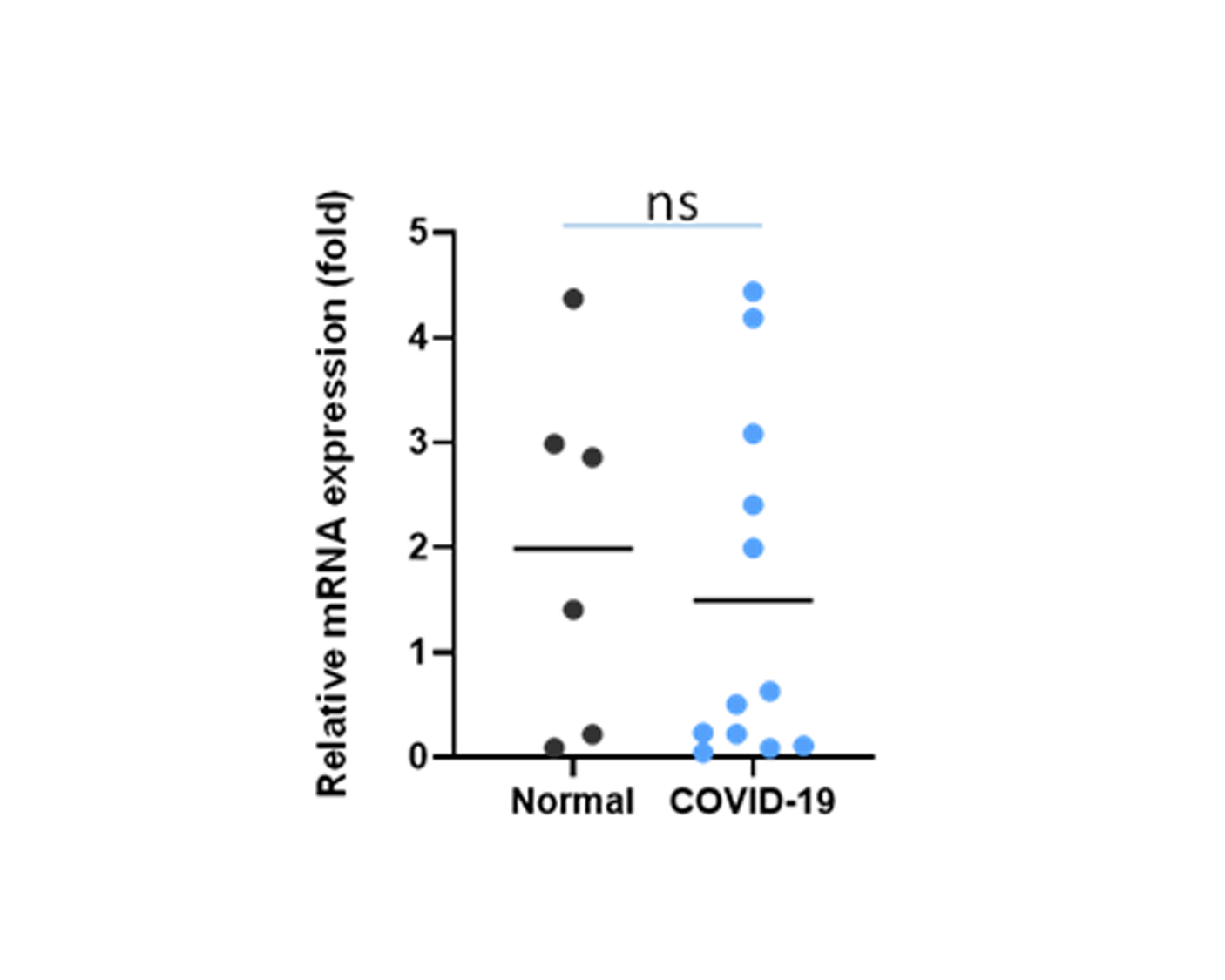

Supplement: FIG S1 [file mbio.00951-22-s0002.tif]

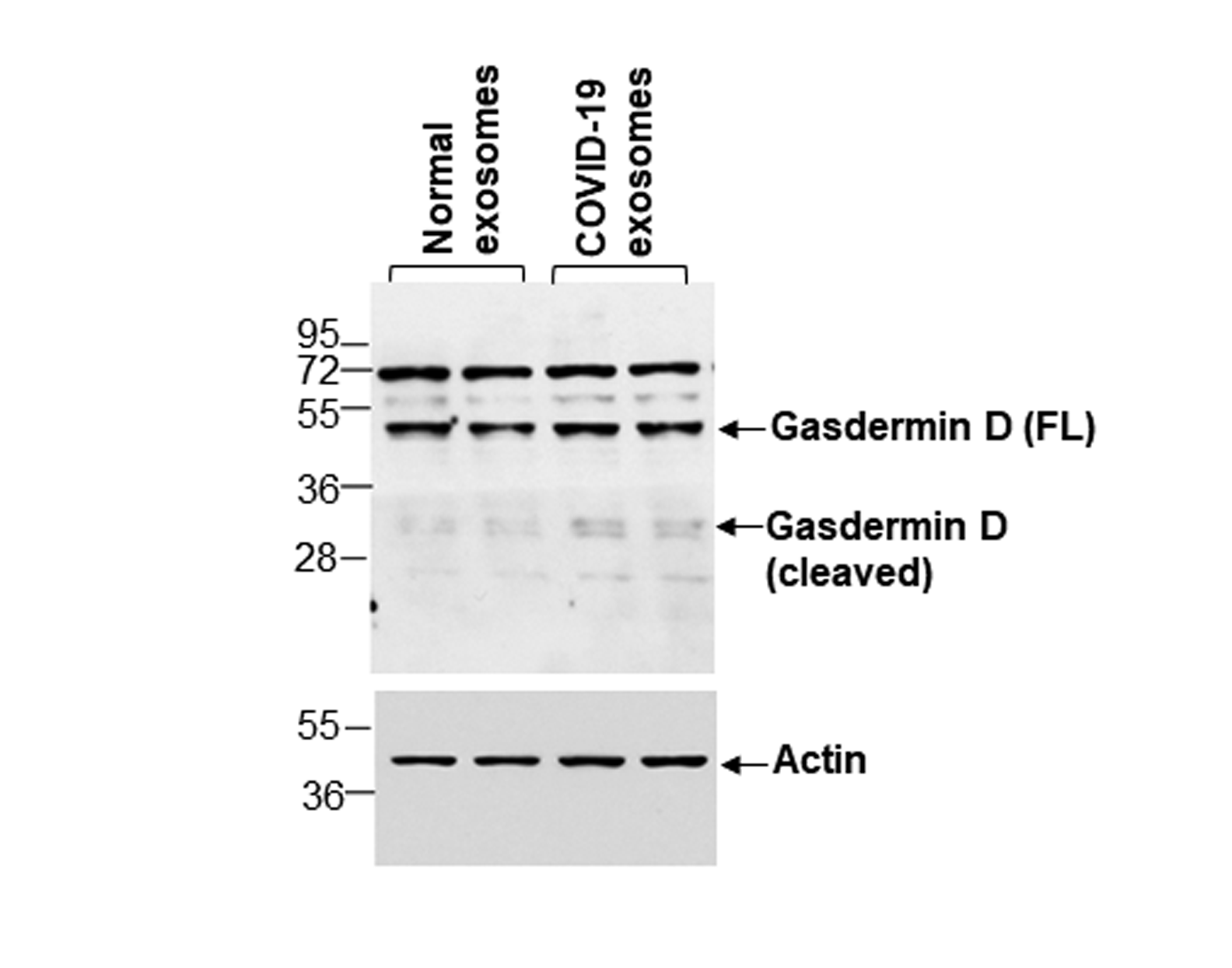

Supplement: FIG S2 [file mbio.00951-22-s0003.tif]
