# Supplementary material for: Circulatory Exosomes from COVID-19 Patients Trigger NLRP3 Inflammasome in Endothelial Cells
Source: mBio. 2022 May 19;13(3):e00951-22. doi: 10.1128/mbio.00951-22 (PMC9239151; doi:10.1128/mbio.00951-22)
Supplement: TEXT S1 [file mbio.00951-22-s0001.pdf]

Supplementary Document

Circulatory exosomes from COVID-19 patients trigger NLRP3 inflammasome in endothelial cells

Subhayan Sur[1], Robert Steele[1], T. Scott Isbell[1], Ranjit Ray[2], Ratna B. Ray[1*]

Departments of Pathology[1], and Internal Medicine[2], Saint Louis University, Missouri, USA

Material and Methods:

Patient Sample: We obtained a total 20 plasma samples of COVID-19 patients admitted at ICU from the Saint Louis University hospital as reported previously (Sur et al., 2021). Best of our knowledge, these patients are not treated with any steroids when the samples were collected. We recently obtained 5 plasma samples from mild symptom of COVID-19 patients. We included plasma samples from 6 healthy volunteers as normal control. The study was approved by the Saint Louis University Institutional Review Board. All the subjects provided signed informed consent for the study.

Institutional Review Board Statement: This study was waived by the Saint Louis University Institutional Review Board for use of deidentified clinical specimens.

Exosomes isolation: The exosomes from the patient or normal samples were isolated using the ME Kit (ME-020p-Kit, New England Peptide Inc, MA) according to the manufacturer's instruction as described previously (Sur et al., 2021).

Cell culture and treatment: Immortalized human microvascular endothelial cells HMEC1 (obtained from ATCC), human-liver endothelial cells TMNK-1 and human monocytic THP-1 were cultured in Dulbecco's modified Eagle's medium (DMEM; Sigma) containing 10% fetal bovine serum (FBS; Sigma), 100 U of penicillin/ml, and 100 μg of streptomycin/ml (Sigma). Cells were incubated with exosomes for 2 hr and then complete medium- was added. The cells were harvested after 48 hr of incubation for further analysis. Exosome depleted serum (Invitrogen) containing media was used as negative control.

RNA isolation and expression analysis: Total RNA was isolated using TRIzol reagent (Invitrogen, CA). The cDNA was synthesized using a random hexamer with SuperscriptIII reverse transcriptase (ThermoFisher Scientific). Real-time PCR was performed for quantitation of gene expression using TaqMan Universal PCR master mix and 6-carboxyfluorescein (FAM)-MGB probes for NLRP3 (assay ID: Hs00918082_m1), CASP1 (assay ID: Hs00354836_m1) and IL-1β (assay ID: Hs01555410_m1) as per manufacture's protocol (Thermo Fisher Scientific). 18s (assay ID: Hs03928985_g1) was used as endogenous control. The relative gene expression was analyzed by $2^{-\Delta\Delta CT}$ formula ($\Delta\Delta CT = \Delta CT$ of the sample − ΔCT of the control). Each sample was loaded in triplicate.

Western blot analysis:

Lysates were analyzed by western blot using specific antibody to caspase-1 (Cell Signaling Technology, CST) followed by incubation with HRP-conjugated anti-rabbit secondary antibody

(Bio-Rad). The blot was reprobed with Actin- HRP antibody (Santa Cruz Biotechnology) to compare protein load in each lane. Densitometry analysis was done using Image J software.

Caspase-1 assay: Caspase-1 activity was measured in culture medium using Caspase-Glo 1 Inflammasome Assay kit (Promega) according to the manufacturer's instruction. In brief, endothelial cells (80,000/ well) were seeded into 12 well plate and treated with exosomes from normal or patientplasmas. Culture media was collected after 48 hr and 50µl of culture media was added into white opaque 96-well microplates. Equal volume Caspase-Glo 1 reagent was added immediately and incubated for 3 hr and the luminescence was measured by Tecan Microplate Reader.

IL-1β ELISA: The IL-1β in culture medium was assayed by ELISA MAX™ Deluxe Set Human IL-1β kit (BioLegend) according to the manufacturer's instructions. In brief, 100 µl culture media of exosomes from normal or COVID-19 patients were exposed to endothelial cells, added into IL-1β antibody coated 96 well high binding ELISA microplate and incubated for 2 hr. After washing, IL-1β detection antibody was added for 1 hr at room temperature followed by incubation with Avidin-HRP solution and substrate. The reaction was stopped by 2N H_2SO_4 and absorbance was measured at 450 nm. A IL-1β standard was serially diluted and processed similarly to quantify the concentration of IL-1β in experimental sample from the standard curve.

Statistical Analysis: The results were expressed as means ± the standard error. A two tailed Student's t test was used for comparisons between the groups (normal vs. COVID-19 exosome

treated samples or mild vs severe samples). P-value less than 0.05 was considered statistically significant. All experiments were repeated at least three times, and representative data are shown.

Reference:

Sur S, Khatun M, Steele R, Isbell TS, Ray R, Ray RB. 2021. Exosomes from COVID-19 Patients Carry Tenascin-C and Fibrinogen-beta in Triggering Inflammatory Signals in Cells of Distant Organ. Int J Mol Sci 22.
